# Accurate interpretation of thyroid dysfunction during pregnancy: should we continue to use published guidelines instead of population-based gestation-specific reference intervals for the thyroid-stimulating hormone (TSH)?

**DOI:** 10.1186/s12884-022-04608-z

**Published:** 2022-03-31

**Authors:** Rana Turkal, Cem Armağan Turan, Onur Elbasan, Serenay Aytan, Burcu Çakmak, Büşra Gözaydınoğlu, Duygu Ceyda Takır, Ozan Ünlü, Günel Bahramzada, Ahmet Faruk Tekin, Tülay Çevlik, Esra Esim Büyükbayrak, Önder Şirikçi, Hülya Gözü, Goncagül Haklar

**Affiliations:** 1 Biochemistry Laboratory, Marmara University Pendik Education and Research Hospital, Istanbul, Turkey; 2 Department of Internal Medicine, School of Medicine, Marmara University, Istanbul, Turkey; 3 Subdepartment of Endocrinology, Department of Internal Medicine, School of Medicine, Marmara University, Istanbul, Turkey; 4 Medical Student, School of Medicine, Marmara University, Istanbul, Turkey; 5 Department of Biochemistry, School of Medicine, Marmara University, Istanbul, Turkey; 6 Department of Gynecology and Obstetrics, School of Medicine, Marmara University, Istanbul, Turkey

**Keywords:** Thyroid dysfunction, Pregnancy, Gestation-specific, Reference interval, ATA

## Abstract

**Background:**

Considering the changes in thyroid physiology associated with pregnancy and poor outcomes related to abnormal maternal thyroid function, international guidelines recommend using population-based trimester-specific reference intervals (RIs) for thyroid testing. If these RIs are not available in the laboratory, implementing recommended fixed cut-off values globally is still controversial. To address this issue, we aimed to establish appropriate RI of thyroid-stimulating hormone (TSH) in pregnant Turkish women for our laboratory and compare the prevalence of thyroid dysfunction based on the established and recommended criteria.

**Methods:**

Of 2638 pregnant women, 1777 women followed in the obstetric outpatient were enrolled in the reference interval study after applying exclusion criteria related to medical and prenatal history. A retrospective study was conducted by collecting data from July 2016 to March 2019. Serum TSH was measured by UniCel DxI 800 Immunoassay System (Beckman Coulter Inc., Brea, CA, USA). The study design relied on two approaches in order to classify pregnant women: trimester-specific and subgroup-specific; the latter involved dividing each trimester into two subgroups: T1_a_, T1_b_, T2_a_, T2_b_, T3_a_, T3_b_. The lower and upper limits of the RIs were derived by the parametric method after normalizing the data distribution using the modified Box-Cox power transformation method.

**Results:**

The lowest TSH value was detected at 8-12 weeks in early pregnancy, and the median value of TSH in the T1_b_ subgroup was significantly lower than the T1_a_ subgroup (*P* < 0.05). TSH levels showed a gradual trend of increase along with the pregnancy and increased significantly in the T2_a_, T2_b,_ and T3_b_ subgroups compared to the preceding subgroups (*P* < 0.05). Compared to the diagnostic criteria recommended by American Thyroid Association (ATA), the prevalence of thyroid dysfunction was significantly different from the established trimester- and subgroup-specific RIs throughout the pregnancy (*P* < 0.001).

**Conclusions:**

We conclude that establishing gestation- and laboratory-specific RIs, especially for TSH, is essential for diagnosing thyroid disorders in pregnancy, and the recommended universal cut-off values, which may contribute to the risk of a misdiagnosis or a missed diagnosis, should be taken with caution in the clinical setting. However, regarding the fluctuation of thyroid function tests throughout pregnancy, trimester-specific RIs are insufficient, and implementing split phases is required.

## Background

Pregnancy is accompanied by various physiological alterations that have an impact on the thyroid environment, involving an increase in renal iodine clearance, estrogen-induced rise in serum thyroxine-binding globulin (TBG), thyroid stimulation by human chorionic gonadotropin (hCG), and increased thyroid hormone production [1]. Dynamic variation of serum hCG levels and physiological adaptations of the thyroid gland during pregnancy influence the thyroid function test results [1, 2]. Gestational thyroid dysfunction is common, with a prevalence of 2–4% [3, 4]. Moreover, thyroid dysfunction may result in obstetric complications and irreversible effects on the fetus, including preeclampsia, abruption placenta, spontaneous abortion, low birth weight, and prematurity [5–7]. Considering the changes in thyroid physiology associated with pregnancy and poor outcomes related to abnormal thyroid function, it is essential to establish reference intervals (RIs) for thyroid hormones in pregnant women.

National guidelines throughout the world have also recommended using population-based trimester-specific RIs for thyroid testing, derived from local population data representing a provider’s laboratory due to ethnic differences and geographical variations in populations [2, 8]. There has been a considerable amount of data documenting population-based gestational RIs since these guideline statements. Although the studies reveal significant variation in the reference limit values for TSH, mostly these fixed RIs recommended by the American Thyroid Association (ATA) and the American Endocrine Association are still in use: first trimester, 0.1–2.5 mIU/L; second trimester, 0.2–3.0 mIU/L; third trimester, 0.3–3.0 mIU/L [9, 10]. In addition, the 2017 ATA guideline updated the recommendation by suggesting a new threshold of 4 mIU/L for the TSH upper reference limit in early pregnancy [2]. However, whether these reference ranges should be used globally is still a matter of debate. To address this issue, we aimed to establish gestation- and laboratory-specific RIs for TSH and determine its trend throughout the pregnancy. Our second aim was to use a more comprehensive approach to establish RIs and subsequently compare the prevalence of thyroid dysfunction based on the newly established reference ranges and ATA guidelines recommendation.

## Methods

**Fig. 1:**
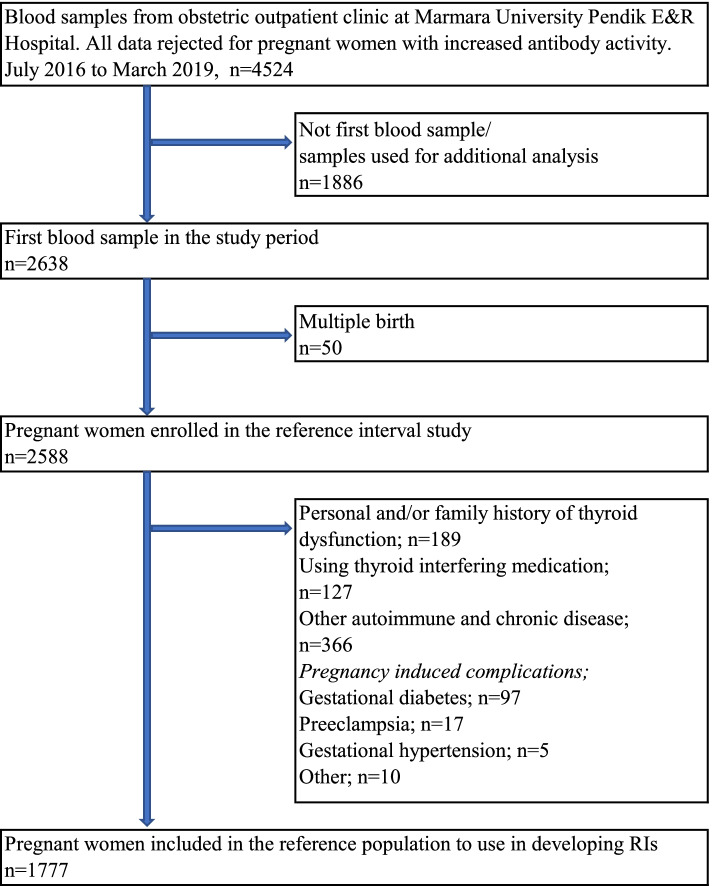
Flow chart of included/excluded pregnant women

The study group included pregnant women visiting the obstetric outpatient clinic at Marmara University Pendik Education and Research Hospital in Turkey in 2016–2019. A retrospective study was conducted by collecting data from July 2016 to March 2019. We obtained detailed information from medical records, consisting of demographic, medical, and obstetrics history, thyroid function tests, and treatment details. We rejected all data for pregnant women with positive anti-thyroid peroxidase (Anti-TPO) and/or anti-thyroid globulin (Anti-TG). Our laboratory reference values (95th percentile) were < 9 IU/mL for TPOAb, and < 4 IU/mL for TgAb. Two thousand six hundred thirty-eight pregnant women (n:2638) contributing to 4524 serum samples participated in the study (Fig. 1). By filtering for the repeated values throughout the pregnancy per pregnant woman, we ensured that only the first result of these pregnant women was used to calculate reference limits [11]*.* The women with multiple pregnancies were removed from the data analysis, leaving 2588 pregnant women in the reference interval study. Gestational ages were estimated by an ultrasound examination and the last menstrual period. Exclusion criteria for defining the appropriate reference population used in estimating the reference range for TSH comprised the suggestions of the National Academy of Clinical Biochemistry (NACB): family or personal history of thyroid disease (hypo- or hyperthyroidism, thyroid cancer, visible or palpable goiter) and using medications including thyroid hormone (e.g., levothyroxine) or antithyroid drugs (e.g., methimazole or propylthiouracil) or any drug that might affect thyroid function tests such as dopamine, glucocorticoids, anticonvulsants, salicylates, lithium carbonate, iodine-containing prescriptions and several others [12–14]. Additionally, women who had any chronic/autoimmune disease (e.g., hypertension, diabetes, rheumatoid arthritis, psoriasis, and cancer) and pregnancy complications (e.g., gestational diabetes, preeclampsia, gestational hypertension, spontaneous abortion) were further ruled out. Of 2638 pregnant women, 1777 women were enrolled in the reference interval study after applying exclusion criteria related to medical and prenatal history. For the assessment of maternal thyroid dysfunction, the entire cohort was used. To preserve confidentiality, we coded each patient and removed their original identifications. The study was approved by the Marmara University Faculty of Medicine Research Ethics Committee (protocol code: 09.2018.811), which waived the requirement for informed consent due to the retrospective design of the study and anonymous nature of the data collection process.

Serum levels of TSH were measured by Unicel DxI 800 immunoassay analyzer (Beckman Coulter Inc., CA, USA), which uses chemiluminescent technology for quantitative analysis. The assay manufacturer’s reference interval was 0.34–5.6 mIU/L, and the inter-assay imprecision (CV%) for two levels of internal quality control (QC) material used for TSH was 4.9 and 5.7% at concentrations of 2.82 and 9.21 mIU/L, respectively. The inter-assay imprecision of TSH was determined by running two levels of QC material once a day over 20 days on two separate analyzers.

### Statistical analysis

MedCalc® Statistical Software version 19.6 (MedCalc Software Ltd., Ostend, Belgium) and IBM SPSS Statistics version 23.0 (IBM Corp, Armonk, NY, USA) were used for the analysis. Reference population data were examined by nonparametric trend curves [locally estimated scatter plot smoothing (LOESS)] to visually assess the relationship between the gestational age and the TSH concentrations. The normality of the data distribution was assessed through Kolmogorov–Smirnov method. We performed the Tukey test to remove outliers. The lower and upper limits (LL and UL) of the reference intervals were determined by the parametric method after transforming the data to a normal distribution with the modified Box-Cox power transformation method, and 90% confidence intervals (CIs) for the LL and UL of each reference interval were calculated [15]. Scatter plots of gestational age against TSH concentrations were generated to visually compare the established and the recommended reference intervals. Mann-Whitney U-test (numerical variables) and χ2 or Fisher’s exact test (categorical variables) were used to compare the variables between two groups. *P* < 0.05 was considered significant. The level of agreement between the different reference ranges to evaluate the thyroid function of pregnant women was quantified by Kappa value (κ). Adopting the interpretative assessment of kappa consistency outlined by McHugh, concordance was classified as minimal (0.21–0.39), weak (0.40–0.59), moderate (0.60–0.79), and strong (0.80–0.90) [16].

## Results

The study design relied on two approaches in order to classify pregnant women. The first approach (presented as trimester-specific) involved categorizing according to trimesters: T1 (1-12 weeks), T2 (13-25 weeks), and T3(26-41 weeks). The second approach (presented as six subgroups) involved classifying each T1, T2, and T3 trimesters into two subgroups: T1_a_, T1_b_, T2_a_, T2_b_, T3_a_, T3_b_. Demographic data concerning women in these groups are shown in Table 1.

**Table 1:** Demographic details for the reference interval study population

Group	No	Maternal age, years*	Gestational age, weeks*
T1	705	28 (16-46)	8.6 (1.3-12.6)
T1_a_	245	28 (16-45)	6.6 (1.3-7.6)
T1_b_	460	28 (16-46)	10 (8-12.6)
T2	692	28 (17-44)	18.1 (13-25.6)
T2_a_	453	28 (17-43)	16.4 (13-19.6)
T2_b_	239	28 (17-44)	22.4 (20-25.6)
T3	380	27 (16-47)	31.5 (26-40.3)
T3_a_	228	28 (17-43)	29.3 (26-32.6)
T3_b_	152	27 (16-47)	35 (33-40.3)

*Values are expressed as median (range)

The median maternal age in the reference population was 28 years (range:16–47 years). In the first trimester, for a total of 705 women, the median maternal and gestational age was 28 years and 8.6 weeks, respectively. In the second trimester, for a total of 692 women, median maternal and gestational ages were 28 years and 18.1 weeks, respectively. In the third trimester, for a total of 380 women, the median maternal age was 27 years, and the median gestational age was 31.5 weeks (Table 1).

### Reference intervals of TSH for the three trimesters and six subgroups

The TSH reference range in the first, second and third trimesters was 0.23 to 3.09 mIU/L, 0.54 to 3.01 mIU/L, 0.66 to 3.23 mIU/L, respectively (Table 2). The upper limits for the TSH reference range were all higher than that of upper reference limits recommended by ATA (2.5 mIU/L for the first trimester, 3 mIU/L in the second and third trimester).

**Table 2:** Trimester-specific reference intervals for TSH (mIU/L)

Trimester	N*	Median	Min-max	Lower limit	Upper limit	Lower 90% CI	Upper 90% CI	*P* value*
T1	670	1.25	0.03-4.47	0.23	3.09	0.19-0.27	2.93-3.24	
T2	666	1.54	1.17-4.07	0.54	3.01	0.49-0.59	2.89-3.12	0.000*
T3	369	1.62	0.31-4.52	0.66	3.23	0.61-0.73	3.05-3.42	0.058

* *P* value was calculated for median TSH values comparing with the upper trimester

As a result of excluding the outliers by the Tukey test in each trimester, the net sample size was slightly different from the one indicated in Table 1

Even though the lowest value of the UL for TSH was detected in the second trimester, the median TSH concentrations of pregnant women in the first trimester significantly decreased compared to that of the second trimester (*P* < 0.05). However, no significant difference in median values was observed between the second and third trimesters (Table 2).

**Fig. 2:**
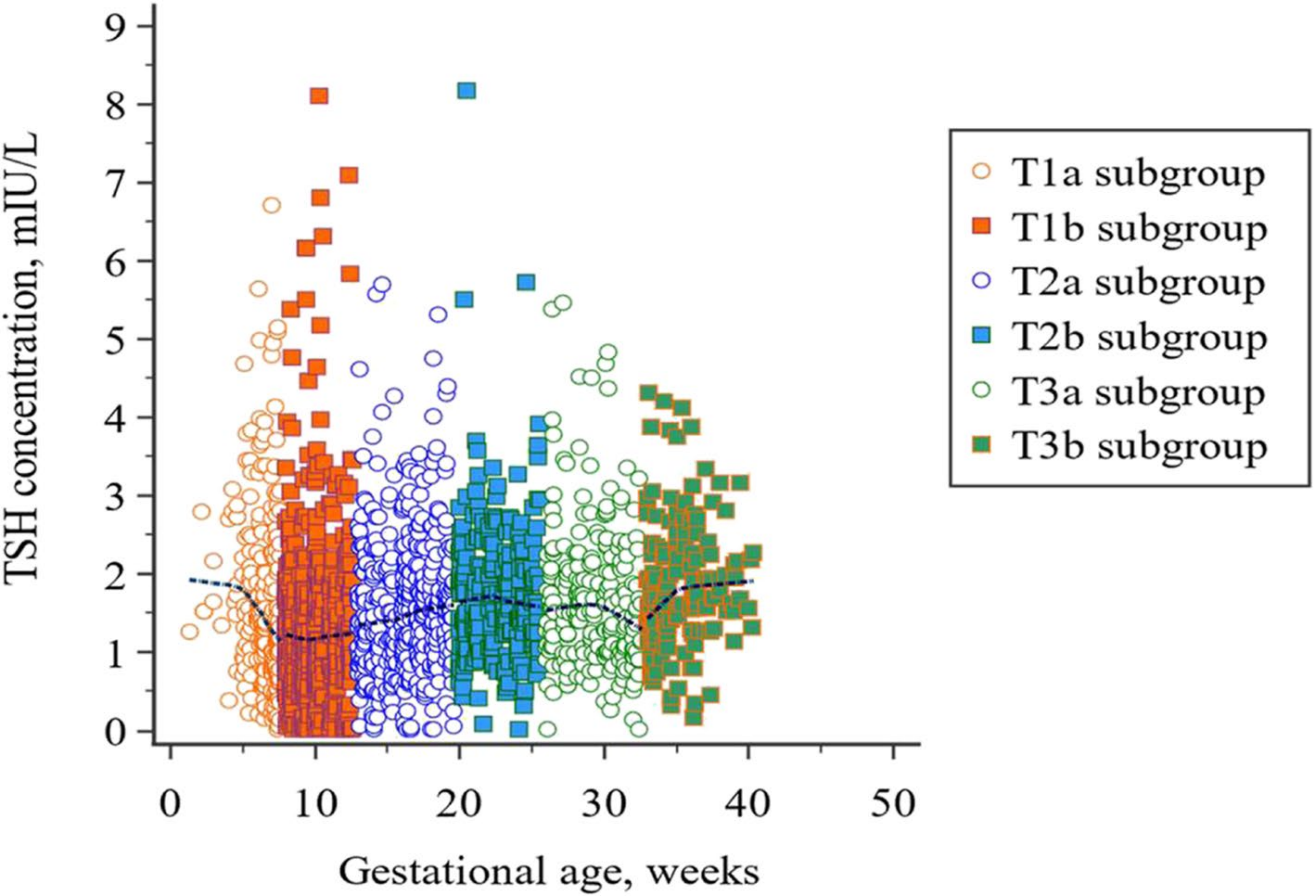
Scatter plot of gestational age versus TSH concentration with a LOESS smoothing line. The first half of the trimesters are represented with round markers and the second half with square markers. Loess nonparametric trend curve is fitted to scatter plots to reveal the trend of TSH levels throughout pregnancy

A scatter plot of gestational age versus TSH concentration with a nonparametric trend line in the reference population is displayed in Fig. 2. The suppressed concentrations of TSH were observed in early pregnancy, followed by a rising trend through the pregnancy.

**Fig. 3:**
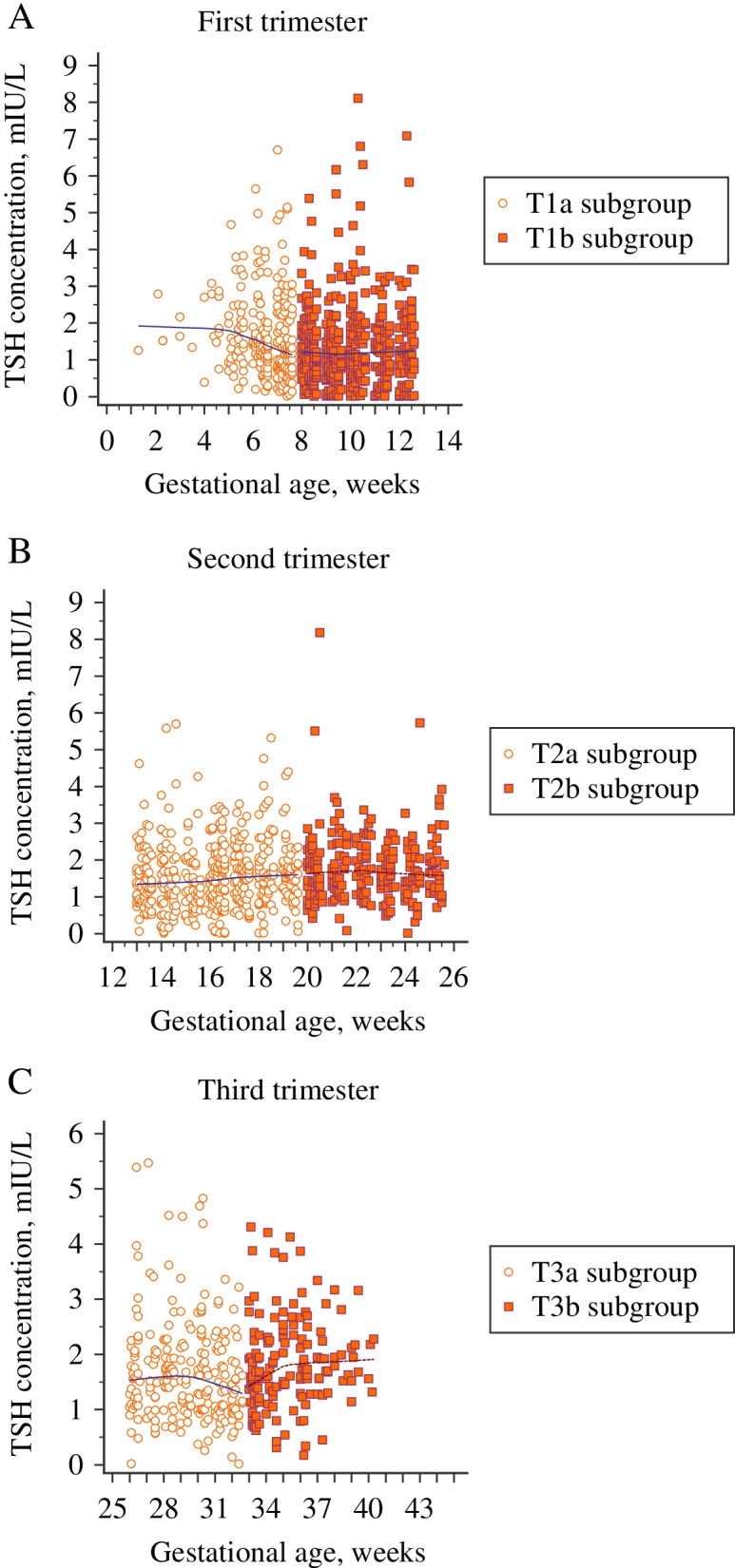
Gestation-specific scatter plots of TSH levels for the three trimesters, including six subgroups. **A** First trimester; **B** Second trimester; **C** Third trimester. The first half of the trimesters are represented with round markers and the second half with square markers. Loess nonparametric trend curves (solid lines for the early trimester and dashed lines for the late trimester) are fitted to scatter plots to reveal the trend of TSH levels throughout the six subgroups

Six subgroup-specific reference intervals are shown in Table 3. Each trimester was split into two separate subgroups to demonstrate the varying patterns of TSH levels throughout the pregnancy. The median TSH value of each subgroup is considered significantly different from that of the upper subgroup when P is less than 0.05. Gestation-specific scatter plots of TSH with nonparametric trend curves for the three trimesters, including six subgroups in the reference population, are also visualized in Fig. 3. A remarkable decline in TSH levels during the second half of the first trimester was confirmed by Fig. 3A. The TSH concentrations increased significantly in the T2a and T2b subgroups compared to the upper subgroup (*P* < 0.05). However, the median value in subgroup T3a decreased compared to that of the T2b subgroup, though no significant difference was observed in TSH concentrations between these two subgroups. TSH concentrations showed a gradual rise again from the middle of the third trimester to the last stages of pregnancy (Fig. 3C). The median TSH value in subgroup T3b was significantly higher compared to that in subgroup T3a (*P* < 0.05).

**Table 3:** 6 subgroup reference intervals for TSH

Subgroup*	N	Median	Min-max	Lower limit	Upper limit	Lower 90% CI	Upper 90% CI	*P* value*
T1_a_	242	1.35	0.14-5.65	0.34	3.74	0.28-0.41	3.40-4.11	
T1_b_	448	1.13	0.015-4.47	0.12	2.97	0.08-0.16	2.78-3.16	0.000*
T2_a_	432	1.51	0.16-4.07	0.48	3.00	0.42-0.54	2.85-3.15	0.000*
T2_b_	233	1.61	0.32-3.92	0.68	3.02	0.60-0.77	2.84-3.20	0.009*
T3_a_	220	1.53	0.37-4.52	0.65	3.17	0.59-0.73	2.91-3.44	0.187
T3_b_	145	1.7	0.54-4.13	0.82	3.20	0.72-0.92	2.95-3.47	0.008*

*As a result of different number of outliers excluded in each subgroup, net sample size was slightly different for each trimester and subgroup

We also evaluated thyroid dysfunction, including hypothyroidism and hyperthyroidism, using RIs established in the three trimesters and six subgroups compared to the RIs proposed by ATA guidelines. The frequencies of each thyroid dysfunction are summarized in Tables 4 and 5.

**Table 4:** Comparison between ATA and trimester-specific reference intervals

Trimester	No. of subjects	Prevalence of thyroid dysfunction based on two criteria, n (%)		Prevalence of hyporthyroidism based on two criteria, n (%)		Prevalence of hyperthyroidism based on two criteria, n (%)		Kappa value	*P* value
		Trimester-specific^a^	ATA guidelines^b^	Trimester specific^a^	ATA guidelines^b^	Trimester-specific^a^	ATA guidelines^b^		
First	705	116 (16.5)	134 (19.0)	60 (8.5)	102 (14.5)	56 (7.9)	32 (4.5)	0.69	< 0.001
Second	692	94 (13.6)	62 (9.0)	43 (6.2)	44 (6.4)	51 (7.4)	18 (2.6)	0.76	< 0.001
Third	380	44 (11.6)	34 (8.9)	22 (5.8)	29 (7.6)	22 (5.8)	5 (1.3)	0.66	< 0.001

*TSH* Thyroid stimulating hormone, *ATA* American Thyroid Association

^a^Trimester-specific reference values for TSH in pregnant women are defined as the lower and upper limits derived by the parametric method after normalizing the data distribution using the modified Box-Cox power transformation method: 0.23 to 3.09 mIU/L in the first trimester, 0.54 to 3.01 mIU/L in the second trimester, 0.66 to 3.23 mIU/L in the third trimester. ^b^Guidelines from the 2011 ATA recommendation maternal TSH between 0.1 to 2.5 mIU/L in the first trimester, 0.2 to 3.0 mIU/L in the second trimester, and 0.3 to 3.0 mIU/L in the third trimester

**Table 5:** Comparison between ATA and 6 subgroup-specific reference intervals

Subgroup	No. of subjects	Prevalence of thyroid dysfunction based on two criteria, n (%)		Prevalence of hyporthyroidism based on two criteria, n (%)		Prevalence of hyperthyroidism based on two criteria, n (%)		Kappa value	*P* value
		Subgroup-specific^a^	ATA guidelines^b^	Subgroup-specific^a^	ATA guidelines^b^	Subgroup-specific^a^	ATA guidelines^b^		
T1_a_	245	29 (11.8)	49 (20.0)	14 (5.7)	47 (19.2)	15 (6.1)	2 (0.8)	0.33	< 0.001
T1_b_	460	69 (15.0)	85 (18.5)	37 (8.0)	55 (12)	32 (7.0)	30 (6.5)	0.85	< 0.001
T2_a_	453	61 (13.5)	47 (10.4)	31 (6.8)	31 (6.8)	30 (6.6)	16 (3.5)	0.85	< 0.001
T2_b_	239	25 (10.5)	15 (6.3)	13 (5.4)	13 (5.4)	12 (5.0)	2 (0.8)	0.73	< 0.001
T3_a_	228	30 (13.2)	21 (9.2)	15 (6.6)	17 (7.5)	15 (6.6)	4 (1.8)	0.72	< 0.001
T3_b_	152	25 (16.4)	13 (8.6)	8 (5.3)	12 (7.9)	17 (11.2)	1 (0.7)	0.43	< 0.001

*TSH* thyroid stimulating hormone, *ATA* American Thyroid Association

^a^Subgroup-specific reference values for TSH in pregnant women are defined as the lower and upper limits derived by the parametric method after normalizing the data distribution using the modified Box-Cox power transformation method: 0.34 to 3.74 mIU/L in the T1_a_ subgroup, 0.12 to 2.97 mIU/L in the T1_b_ subgroup, 0.48 to 3.00mIU/L in the T2_a_ subgroup, 0.68 to 3.02 mIU/L in the T2_b_ subgroup, 0.65 to 3.17 mIU/L in the T3_a_ subgroup and 0.82 to 3.20 mIU/L in the T3_b_ subgroup

^b^Guidelines from the 2011 ATA recommendation maternal TSH between 0.1 to 2.5 mIU/L in the first trimester, 0.2 to 3.0 mIU/L in the second trimester, and 0.3 to 3.0 mIU/L in the third trimester

### Comparison between ATA and trimester-specific reference ranges

**Fig. 4:**
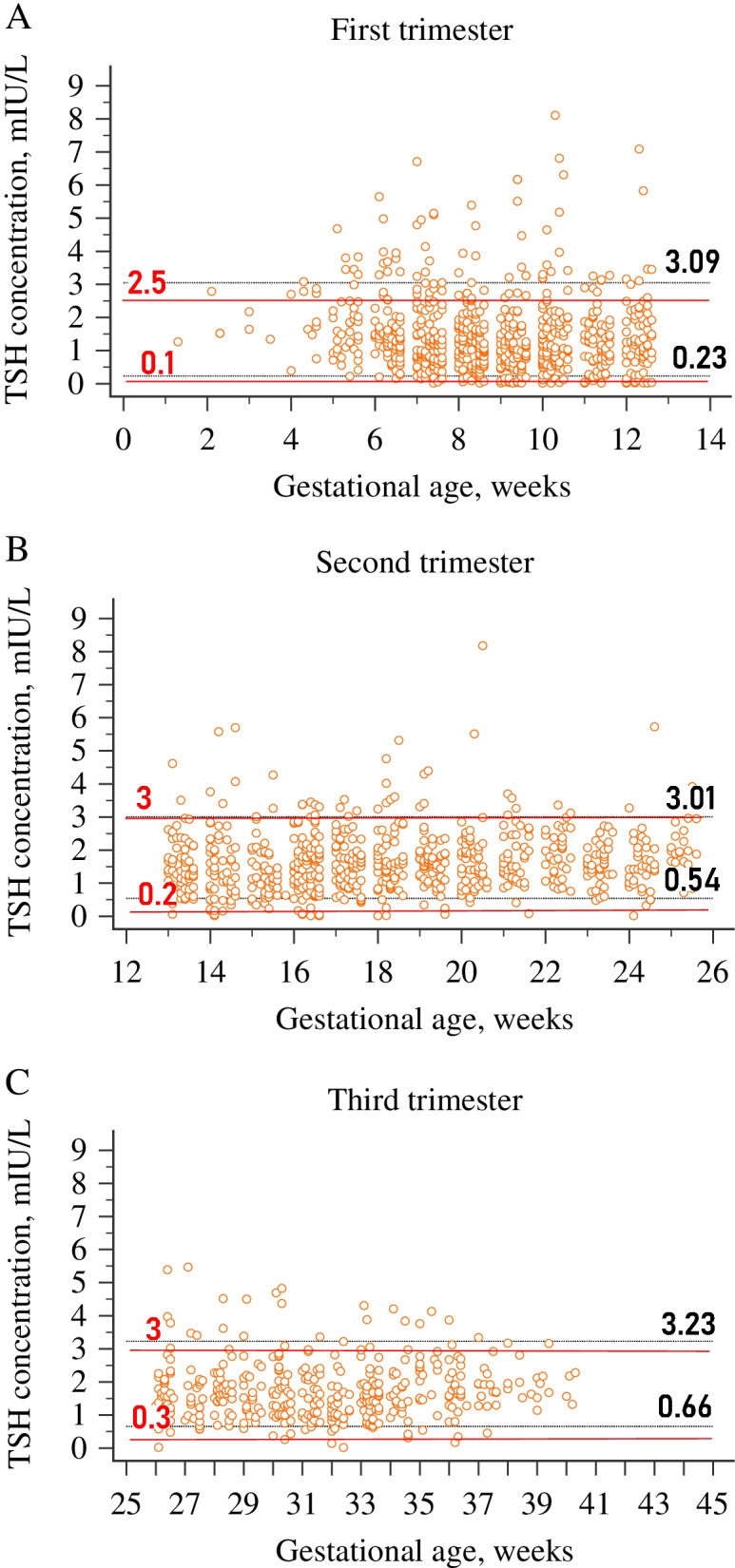
Comparison between ATA and trimester-specific reference intervals. **A** First trimester; **B** Second trimester; **C** Third trimester. The figure illustrates the gaps between estimated and fixed cut-off values. For all graphs, the solid red lines are the upper and lower reference limits suggested by the ATA 2011. The dotted black lines are the upper and the lower reference limits derived from 1777 healthy pregnant women in our laboratory for each trimester

Compared to the diagnostic criteria recommended by ATA, the prevalence of thyroid dysfunction was significantly higher by the trimester-specific reference range in the second and third trimester (13.6% vs. 9 and 11.6% vs. 8.9%; *P* < 0.001 respectively); however, it was lower in the first trimester (16.5% vs. 19.0%, *P* < 0.001). Using the ATA guidelines for pregnant women in our population, 102 women (14.5%) in the first trimester, 44 (6.4%) in the second trimester, and 29 (7.6%) in the third trimester met the criteria for hypothyroidism, while based on trimester-specific reference range, 60 (8.5%), 43 (6.2%), and 22 (5.8%) pregnant women were detected as hypothyroidism. On the other hand, applying trimester-based RI, 129 women (21.1%) were detected as hyperthyroidism; of whom 56, 51, and 22 were in the 1st, 2nd, and 3rd trimesters, while only 55 (8.4%) pregnant women presented hyperthyroidism; of whom, 32, 18 and 5 were in the 1st, 2nd, and 3rd trimesters, respectively, if we used ATA criteria. Meanwhile, a moderate level of agreement was obtained between trimester-based RIs and ATA criteria. Comparisons between ATA criteria and trimester-specific reference intervals are illustrated in Fig. 4.

### Comparison between ATA and six subgroup-specific reference ranges

**Fig. 5:**
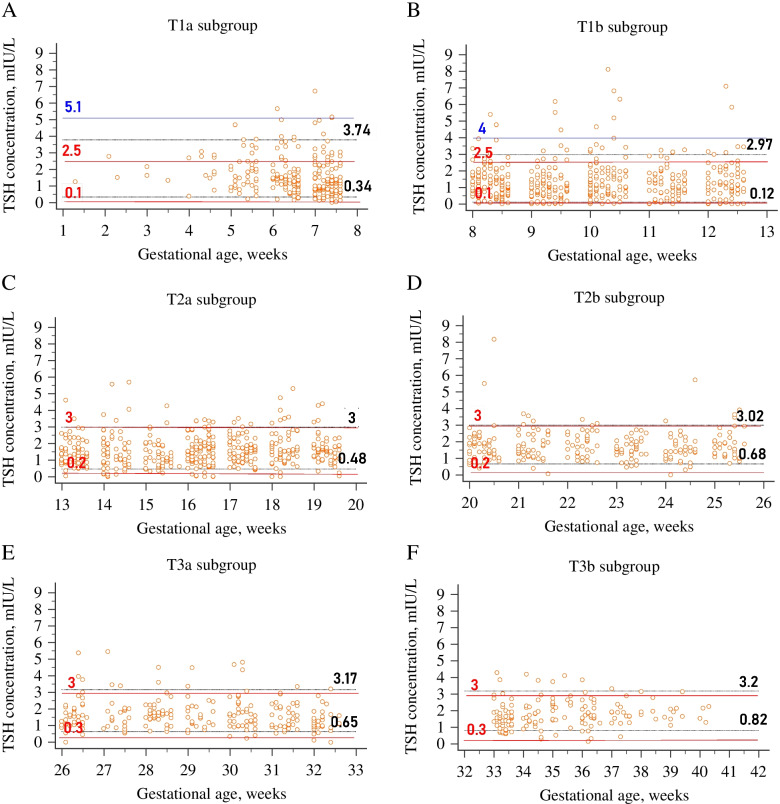
Comparison between ATA and subgroup-specific reference intervals. **A** T1_a_ subgroup; **B** T1_b_ subgroup; **C** T2_a_ subgroup; **D** T2_b_ subgroup; **E** T3_a_ subgroup; **F** T3_b_ subgroup. The figure illustrates the gaps between estimated and fixed cut-off values. For all graphs, the solid red lines are the upper and lower reference limits suggested by the 2011 ATA. The dotted black lines are the upper and the lower reference limits derived from 1777 healthy pregnant women in our laboratory for each subgroup. For the subgroups T1_a_ and T1_b,_ the solid blue line corresponds to the upper reference limit proposed by the 2017 ATA (URL of 5.1 mIU/L, obtained by subtracting 0.5 mIU/L from the non-pregnant upper TSH level for T1_a_ and 4.0 mIU/L for T1_b_ subgroup)

Compared to the diagnostic criteria recommended by ATA, the prevalence of thyroid dysfunction was significantly lower by the subgroup-specific reference range in the T1_a_ and T1_b_ subgroups (11.8% vs. 20.0 and 15.0% vs. 18.5%; *P* < 0.001 respectively); however, it was higher in other subgroups. Significant differences were found in thyroid function evaluation between ATA criteria and the subgroup-specific reference ranges (Table 5). Based on reference ranges suggested by ATA for maternal TSH, the prevalences of hypothyroidism were 19.2 and 12% in subgroups T1_a_ and T1_b_, whereas the prevalences of hypothyroidism decreased to 5.7 and 8.0%, respectively, if the subgroup-specific evaluation was implemented. In addition, a strong level of agreement was observed in the thyroid dysfunction classification between ATA criteria and established RIs in subgroups T1_b_ and T2_a_ (κ-value = 0.85 for both subgroups) while the reference ranges for the T1_a_ and T3_b_ subgroups were minimal in agreement with those of ATA criteria (κ-value = 0.33 and κ-value = 0.43, respectively). In contrast, adopting the 2017 ATA recommendation (URL of 5.1 mIU/L, obtained by reducing the non-pregnant level by 0.5 mIU/L for T1_a_ and 4.0 mIU/L for subgroup T1_b_), the prevalences of hypothyroidism decreased to 1.2 and 2.8%, respectively. Furthermore, when we compared only just the results of the established URL to both previous and revised ATA criteria regarding the concordance of detecting hypothyroidism in the T1_a_ subgroup, the level of agreement was also minimal (κ = 0.40 and κ = 0.34, respectively). However, weak agreement (κ = 0.49) was exhibited between the calculated URL and the 2017 ATA cut-off value in the assessment of hypothyroidism for subgroup T1_b_, while strong agreement (κ = 0.85) remained regarding previous ATA criteria. Comparisons between ATA criteria and subgroup-specific reference intervals are illustrated in Fig. 5.

## Discussion

TSH has been widely accepted as the universal screening tool for thyroid dysfunction in patients, including pregnant women [17]. The physiological log/linear reverse correlation found between serum TSH and free thyroxine (FT4) concentrations and the excellent sensitivity of the pituitary to detect abnormal FT4 values corresponding to the genetically adjusted FT4 setpoint contribute to the superiority of TSH in providing reliable detection of abnormal values within the individual [12]. There are strong arguments implying that commonly available FT4 immunoassays may not work well during pregnancy. Measurement of maternal FT4 by usual techniques poses some difficulties due to the interference of pregnancy-modified plasma proteins, in particular, increased TBG and decreased albumin [18]. Lee et al. highlighted the main challenges regarding maternal FT4 measurements and documented that immunoassays produced results that did not correlate with physiologic FT4 fluctuation observed during pregnancy [19]. Therefore, we have focused our attention, particularly on the TSH reference interval, considering challenging data about the measurement of FT4 during pregnancy. Our population-based RIs for all trimesters and subgroups were higher than the ATA standard. When comparing the TSH reference interval between pregnancy and the manufacturer’s proposed reference limits, upper TSH reference limits showed a noticeable decline during the first trimester. Even though an increase was observed as the pregnancy progressed, cut-off values had not returned to the non-pregnant levels by the following trimesters. The upper and the lower TSH reference limits decreased by 44.8 and 32.3%, respectively, compared with the non-pregnant RI in the first trimester. Lower TSH reference limits also increased throughout pregnancy and reached 1.58 and 1.94 times the non-pregnant level, respectively, at the second and third trimesters. We found higher thresholds compared with the data published by some authors [20, 21] for the URL of TSH in the first and second trimester*,* while higher values were also documented in other studies [22, 23]. Significant variation in URL of TSH has been attributable to population characteristics such as ethnicity in some of these studies [21, 24]. Koreaavar et al. also reported misinterpretation of TSH in 18% of pregnant women due to ethnic discrepancy [25]. In a review analyzing population-based cohort studies on gestational RIs, Medici et al. demonstrated substantial variation in upper TSH reference limits in early pregnancy, ranging from 2.15 mIU/L to 4.68 mIU/L [26]. The review concluded that 90% of these studies documented higher TSH URLs compared with the upper thresholds of 2.5 and 3.0 mIU/L, which are suggested in the guidelines. The clinically significant aspect of this data is that implementing these recommended cut-off values may lead to an overestimation of thyroid dysfunction and overtreatment, resulting in poor pregnancy and fetal outcomes. Overtreatment with levothyroxine, as an extrathyroidal source of the thyroid, may induce thyrotoxicosis [27]. It is also hypothesized that mild thyroid function abnormality compounds the overtreatment risk due to the residual function of the thyroid gland activated by hCG, involving the biological sense process that does not exist between FT4 and hCG [28].

Compared with other studies from Turkey, our data revealed a relatively lower URL for TSH than those reported by Bulur et al. (3.65, 3.63, 3.46 mIU/L in the first, second, and third trimester of pregnancy, respectively) [29]. One thousand two hundred fifty-eight pregnant women were enrolled in this study. Our upper TSH reference limits were also lower than those reported by Akarsu et al. (3.44 and 4.31 mIU/L, respectively), particularly during the second and third trimesters [30]. However, this study (*n* = 2460) reported a lower cut-off value (2.33 mIU/L) for URL of TSH compared with our finding during the first trimester. In his study, TSH levels were measured by Architect i2000SR analyzer (Abbott Diagnostics), and this discrepancy may be due to the methodology of different manufacturers, as all data were obtained from Turkish women without ethnic differences. Although we demonstrated an increasing trend in the median of TSH from the first trimester to the third trimester, which is compatible with the data of these two studies from Turkey, no significant change in TSH median values was observed between the second and third trimester (*P* = 0.058). However, Akarsu et al. also found a significant difference between the second and third trimesters (*P* < 0.001).

We observed that implementing trimester-based RIs instead of using fixed cut-off value could reduce the thyroid dysfunction prevalence from 19 to 16.5% in the first trimester, improving the clinical assessment of 13% of women who were considered to have abnormal thyroid function. Applying the recommended 2.5 mIU/l as the URL for TSH in the first trimester, 14.5% of the pregnant women would present hypothyroidism, versus 8.5% when adopting the established RIs based on trimesters. A study embedded in a population-based cohort reported that 8.6% of the study population with normal TSH concentrations presented levels greater than the recommended threshold in the first trimester, while 4.9% in the second trimester [31].

Comparability of the data concerning thresholds for thyroid function testing is hampered by using many varying methodologies to determine reference limits. Nevertheless, in the context of ATA recommendations, the guideline does not propose any method- or instrument-specific reference ranges for serum TSH. Springer et al. compared maternal thyroid hormone RIs established with seven different analytical systems [32]. Higher reference range limits of TSH (0.25–3.86 mIU/L) were suggested for Modular E170 (Roche Diagnostics) and IRMA Immunotech (Beckman Coulter), while lower limits (0.17–2.81 mIU/L) were set for Immulite 2500 (Siemens Healthcare Diagnostics) and AIA 2000 (Tosoh Bioscience). The determined URLs were all lower when compared with those recommended by their manufacturers, which was consistent with our data. The URL of TSH established with DXI 800 (3.33 mIU/L) was slightly higher than the one (3.09 mIU/L) determined with the same analyzer settled in our laboratory. Consequently, the methodological variation could notably affect the establishment of RIs, and the standardization of the analytical issues should be taken into consideration to ensure the comparability of data worldwide.

After fertilization, a trend of elevation is observed in serum TBG and total T4 levels from the 7th week of pregnancy until about week 16, followed by persisting elevated levels throughout the pregnancy [33]. Direct hCG stimulation on the TSH receptor induces the decline of TSH levels in response to the increased production of thyroid hormones. The most significant reduction in TSH concentration is detected in the first trimester since the peak hCG concentration is reached by around the 10-12 weeks of pregnancy. Dividing each trimester into two groups reflected stage-dependent TSH fluctuations in pregnant women. Since the lowest value of TSH was demonstrated at 8-12 weeks in the second part of the first trimester and the median value of TSH in the T1_b_ subgroup decreased significantly compared to the value in the T1_a_ subgroup (*P* < 0.05), gradual physiologic adjustment throughout the pregnancy is promptly recognized (Fig. 2). Dashe et al. also evaluated varying TSH concentrations of 13,599 pregnant women for each gestational week [34]. They noted the lowest value at the 10th week, accompanied by a gradual rise to the end of the third trimester. A Chinese study including 4800 women reported that TSH levels fell significantly to trough at the 10-11th week, beginning to decline from the 7th week of gestation [35]. In the former research, the highest hCG value was observed at the 9-11th gestational weeks, which concurred with the nadir in TSH. Similar to our study, they demonstrated a lower median concentration of TSH in the late first trimester compared to the early gestational weeks. Though the pattern of decreasing TSH trend observed in our study during the first trimester was compatible with the findings of these studies, we found the suppression of TSH at earlier gestational weeks in the first trimester. Recruiting pregnant women as early as the beginning of the pregnancy allowed sufficient data samples for our analyses in the context of the early gestational TSH concentrations associated with physiological variations in hCG. This remarkable decline in the second half enables us to clarify the notable gap for the URL of TSH (2.3 and 4.5 mIU/L) in the first trimester between two published papers concerning similar populations [36, 37]. The most probable reason for this reported disparity is the various gestational age of selection since one of the papers comprised a limited number of pregnant women before the 8th week of gestation, which may lead to undervaluing the reference limits in the first trimester [36]. In addition, Li et al. concluded that application of TSH RIs established from 7 to 12th weeks of gestation would cause a 5.5% misdiagnosis of normal pregnant women as subclinical hypothyroidism [35]. At the beginning of the first trimester (< 8 weeks), the lower TSH reference limit (0.34 mIU/L) did not change, while the URL (3.74mIU/L) decreased by 33.2%. Whereas, after 8 weeks, the upper and the lower TSH reference limits (0.12-2.97 mIU/L) decreased by 47 and 64.7%, respectively, compared with the non-pregnant RI. If we adopted only one reference range for the first trimester, some women could be misinterpreted in the early stage of the first trimester because of a lower TSH reference limit based on published guidelines, resulting in overtreatment. In addition, the reference ranges for the T1_a_ and T3_b_ subgroups were low in agreement with those of ATA 2011 criteria, suggesting a significant discrepancy between the ability of established and ATA-based cut-off values to detect thyroid dysfunction. Nevertheless, a strong level of agreement was observed in the thyroid dysfunction classification between ATA 2011 criteria and established RIs in subgroups T1_b_ and T2_a_ (Table 5). A notable finding was that regarding the classification of maternal thyroid dysfunction, the level of agreement between ATA 2011 and trimester-specific RI**s** conflicted by the assessments based on subgroup-specific RIs; while the varying agreement levels were documented for six subgroups (κ values ranging from 0.33 to 0.85), kappa agreement values were moderate for all trimesters. Evaluation of these two statistics verifies that trimester-specific RIs might be unsatisfactory for interpreting TSH values, considering the physiological trend of TSH during pregnancy. Though estimation of trimester-specific RIs for TSH has been stressed for years, recent studies have revealed the significant variation of TSH levels observed, particularly in early pregnancy [38, 39]. Liu et al. presented a similar approach that involved splitting each trimester into two groups to analyze the data [39]. Our results of the divided gestational stage coincide with the current literature. The URL was relatively higher in the preceding subgroup of the third trimester if derived from the total trimester, leading to the potential underdiagnosis of women suffering from hypothyroidism in the early stage of the third trimester. Moreover, we observed that TSH concentrations in T2a, T2b, T3b differ significantly from their preceding subgroups, highlighting the significance of split subgroups.

Regarding ATA 2017 based comparison in the T1a subgroup, a minimal level of agreement (κ = 0.34) was also observed for diagnosing hypothyroidism, similar to the level of agreement found with the previous ATA threshold (κ = 0.40). The discordance between the established and fixed cut-off values in the early first trimester indicates that implementing ATA 2017 criteria may increase the risk of undetected hypothyroid pregnant women while using ATA 2011 recommendation may lead to the misdiagnosis of healthy women as having hypothyroidism. However, high agreement found between ATA 2011 criteria and our estimated RI in the T1b subgroup suggests that previous recommended RIs may be more appropriate for the late first trimester. Gao et al. also reported that adopting ATA 2017 threshold in the first trimester might be a sub-optimal practice for pregnant Chinese women [40].

To guide all patients and clinicians, the 2017 ATA guideline updated the recommendation by reducing the LRL and the URL of TSH by approximately 0.4 mIU/L and 0.5 mIU/L, respectively, in the first trimester when local assessments are not available [2]. Since the URL for TSH has approached 4.0-4.5 mIU/L concentrations from ~ 10 mIU/L over the past years, it was widely reduced by 0.5 mIU/L in the first trimester, corresponding to an URL of 4.0 mIU/L [12]. This cut-off should be primarily implemented beginning from the second half of the first trimester, with a progressive increase in the succeeding trimesters [2]. Therefore, we have used an URL of 5.1 mIU/L (URL of non-pregnant level reduced by 0.5 mIU/L) for the T1_a_ subgroup, corresponding to the early first trimester and 4.0 mIU/L for the T1_b_ subgroup, which represents the late first trimester, considering 2017 ATA criteria. However, when analyzing the 19 studies cited in the 2017 ATA guidelines, only five articles reported an URL of normal ≥4.0 mIU/L. The only one paper reported an upper limit of normal exceeding 3.5 mIU/L, out of the five most extensive reference studies, each comprising more than 5000 pregnant women.

Our local reference population used to determine reference ranges for TSH was also identified, taking into consideration ATA-approved guidelines: all pregnant women with known thyroid disease and positive TPOAb status were excluded from the reference population [2]. However, limitation in our study includes the lack of the iodine status of our study population, though guidelines recommend establishing the RIs in populations with optimal iodine uptake [2, 9, 10, 41]. Whereas, limited reports documenting the apparent influence of iodine status on gestational RIs of thyroid function tests exist. In particular, a Chinese study could not find any impact of low urinary iodine concentrations on TSH levels [42]. Moreover, though there were also further points to be addressed in the clinical context, estimation of method-specific reference intervals for our local pregnant population and the matter on adopting split gestational subgroups rather than trimesters was a focus of this paper, and other clinically relevant issues were left to our forthcoming study.

## Conclusions

Considering the lack of local data regarding the population-based reference intervals for thyroid function tests in pregnant Turkish women, using the reference limits recommended by ATA in 2011 is still a current pattern of practice in our country and the subsequent guideline revised by ATA in 2017, suggesting a new threshold of 4 mIU/L for the TSH upper reference limit in early pregnancy is not adopted for use due to the inconsistent existing reports, which was also verified in the present study.

Our study confirms that the application of the prior version of criteria of ATA to the evaluation of thyroid function in pregnant women could lead to misclassification of patient test results. Therefore, we conclude that establishing gestation- and laboratory-specific RIs, especially for TSH, is essential for diagnosing thyroid disorders in pregnancy, and the recommended universal cut-off values, which may contribute to the risk of a misdiagnosis or a missed diagnosis, should be taken with caution in the clinical setting. However, given the variation of maternal thyroid function tests throughout pregnancy, trimester-specific RIs are insufficient, and implementing split phases is required. Similar to previous researches from Turkey, our data point out the need for well-designed prospective studies to establish reference intervals for thyroid function parameters during pregnancy in Turkish women.

## Data Availability

The dataset analysed during the current study are available from the corresponding author on reasonable request.

## Abbreviations

TBG: Thyroxine-Binding Globulin
hCG: Human Chorionic Gonadotropin
RI: Reference Interval
TSH: Thyroid Stimulating Hormone
ATA: American Thyroid Association
LRL: Lower Reference Limit
URL: Upper Reference Limit
Anti-TPO: Anti-thyroid Peroxidase
Anti-TG: Anti-thyroid Globulin
LL: Lower Limit
UL: Upper Limit
CI: Confidence Interval
FT4: Free Thyroxine
